# Supplementary material for: Risk of adverse pathological features for intermediate risk prostate cancer: Clinical implications for definitive radiation therapy
Source: PLoS One. 2021 Jul 15;16(7):e0253936. doi: 10.1371/journal.pone.0253936 (PMC8281993; doi:10.1371/journal.pone.0253936)
Supplement: S1 Table — (DOCX) [file pone.0253936.s001.docx]

**S1 Table. 95% Confidence interval of Figure 2**

|  | 1. **Upstage** | | 1. **GS Upgrade to >=8** | | 1. **Node Positive Disease** | |
| --- | --- | --- | --- | --- | --- | --- |
|  | Odds Ratio | 95% CI* | Odds Ratio | 95% CI* | Odds Ratio | 95% CI* |
| **GS 3+3/4 : PSA>10 vs ≤10** | 1.19 | 1.00 – 1.43 | 1.41 | 1.02 – 1.97 | 1.27 | 0.89 – 1.83 |
| **GS 4+3:PSA>10 vs ≤ 10** | 1.2 | 0.89 – 1.61 | 1.39 | 0.91 – 2.14 | 1.20 | 0.71 – 2.01 |
| **GS4+3/4: PSA <=10** | 1.32 | 1.10 – 1.58 | 1.96 | 1.40 – 2.74 | 1.45 | 1.00 – 2.10 |
| **GS4+3 vs 3+3: PSA >10** | 1.32 | 0.95 – 1.84 | 1.93 | 1.07 – 3.48 | 1.36 | 0.71 – 2.61 |
| **Every 10-year Increase of Age** | 1.37 | 1.16 – 1.62 | 1.30 | 0.88 – 1.94 | 1.29 | 0.83 – 1.99 |
| **Every 10% Increase of PPC** | 1.31 | 1.24 – 1.39 | 1.06 | 0.94 – 1.19 | 1.25 | 1.11 – 1.41 |
| **Every 1 Core Increase of Total Bx cores** | 1.01 | 0.97 – 1.04 | 0.96 | 0.88 – 1.05 | 0.99 | 0.90 – 1.09 |
