# Supplementary material for: Risk of adverse pathological features for intermediate risk prostate cancer: Clinical implications for definitive radiation therapy
Source: PLoS One. 2021 Jul 15;16(7):e0253936. doi: 10.1371/journal.pone.0253936 (PMC8281993; doi:10.1371/journal.pone.0253936)
Supplement: Attachment — Submitted filename: Response to Reviewers.docx [file pone.0253936.s003.docx]

We are resubmitting the manuscript titled “Risk of adverse pathological features for intermediate risk prostate cancer: clinical implications for definitive radiation therapy”. Based on the reviewers’ constructive input, we have revised our report. We have included the reviewers’ comments below, with our accompanying point-by-point responses in bold font.

Reviewer #1:

1) Consider rewording "Per NCCN guidelines, intermediate risk prostate cancer (PCa) has been defined as having at least one of the following features: cT2b to cT2c, Gleason score (GS) 7 (3+4/4+3), and PSA 10 to 20 ng/ml." as "According to guidelines published by the National Comprehensive Cancer Network (NCCN), intermediate risk prostate cancer (PCa) is defined as having at least one of the following features: cT2b to cT2c, Gleason score (GS) 7 (3+4/4+3), and PSA 10 to 20 ng/ml."

**We have revised the manuscript as suggested.**

2) Consider rewording "For men receiving definitive curative intent radiotherapy (RT), the success of treatment relies on accurately defining the target volume in order to ensure disease control and reduce toxicity. Recent technology improvement in treatment planning and delivery with image guidance and intensity-modulated RT has improved treatment precision by reducing the treatment margins, therefore limiting radiation dose to surrounding normal tissues. However, the reduction of margins without considering the pattern and extent of microscopic spread of disease may negatively influence the outcome." to "For men receiving curative-intent radiotherapy (RT), treatment success relies on accurate delineation of target volumes. Recent technology improvement in treatment planning and delivery with image guidance and intensity-modulated RT has improved treatment precision by reducing the treatment margins, therefore limiting radiation dose to surrounding normal tissues. However, the reduction of margins without considering the pattern and extent of microscopic spread of disease may negatively influence outcomes."

**We have revised the manuscript as suggested.**

Minor comments:
1) Please describe pre-treatment workup in greater detail in the Methods section. In the discussion, it is stated that *none* of the patients underwent MP-MRI prior to treatment. Did pre-treatment work-up solely consist of rectal exam, PSA, and TRUS biopsy/findings? This is crucial to the results of the study.

**Pre-treatment work-up included bone scan and CT of pelvis. Reviewer’s point is well taken and we have added the information in the Methods section.**

2) In the discussion section, I recommend further discussion on the utility of MP-MRI, including rates of upstaging from these studies.

**We appreciate reviewer’s suggestion. We have reviewed the literature and discussed the utility of MP-MRI in prostate cancer staging in the discussion section, including the rates of upgrade/upstage based on one of the most recent study.**

Reviewer #2: A retrospective review addressing a clinically significant question. A Well written, easy read, and statistically sound manuscript. The format of data presentation in figures is right but needs editing/ improvement. The first two figures look busy and difficult to follow. The authors acknowledge the study's limitations and gaps in the data collection, not an uncommon problem in retrospective studies. This will be an excellent addition to the existing literature in the context of uncertainties of clinical staging and delineation of CTV in intermediate-risk prorate cancer patients.

**We appreciate reviewer’s positive comment. We have made changes in Figure 1 and 2 and added a table as a supplement. We hope that they are now clear.**
